# Moving *Geochemical Transactions *forward as an open access journal

**DOI:** 10.1186/1467-4866-7-1

**Published:** 2006-01-01

**Authors:** Martin AA Schoonen, Ken B Anderson, Scott A Wood

**Affiliations:** 1 Department of Geosciences and Center for Environmental Molecular Science, Stony Brook University, Stony Brook, NY, USA; 2 Department of Geology, Southern Illinois University Carbondale, Carbondale, IL, USA; 3 Department of Geological Sciences, University of Idaho, Moscow, ID, USA; 4 Editors-in-Chief

## Editorial

*Geochemical Transactions*, the first online-only journal in geochemistry and environmental chemistry, is now the first major open access journal in this subject area. All issues of Geochemical Transactions, including the back content, will be fully and permanently available online to all, without a subscription charge. Copyright of all future articles will be retained by the authors. Geochemical Transactions remains the official journal of the Division of Geochemistry of the American Chemical Society. The generous support of the Division has made it possible to make the back content available without a subscription charge.

Internet-based open access publishing empowers both authors and readers. As authors you are able to see your work published more quickly and disseminated more widely; you are also in a stronger position to protect your intellectual property rights than in a print world where authors are typically forced to assign copyright to the publisher. As readers you stand to gain access to more research information than ever before. The open access model continues to gain in momentum and is already well established in the life sciences, physics and mathematics. By continuing *Geochemical Transactions *as an open access journal we can now offer the benefits of open access publishing to the geochemical community.

There is good reason to be optimistic about the future of *Geochemical Transactions*. As borne out by its impact factor – 1.92 in 2004 and third among geochemistry journals [1] – *Geochemical Transactions *has rapidly gained respect within the community it serves. However, the subscription model it has been published under has hampered access to the journal and its growth. The open access publishing model [2] removes this barrier. Traditionally, open access journals have been hampered in building up a reputation because it takes several years before a new journal is tracked by ISI and impact factors become available [3,4]. Although impact factors as a tool to rank the impact of journals have their limitations [5], they remain an important factor in the reputation of a journal and its growth. Several open access journals have seen a rapid increase in manuscript submissions once ISI started tracking their content and published their impact factor [3]. Unlike new open access journals, *Geochemical Transactions *is an established journal and is already indexed and tracked by ISI. Under its new publishing model, all future journal content will not only be indexed by ISI but also PubMed [6], Scopus [7], and will be listed in Google Scholar [8]. The combination of open access to all articles and a wider indexing is expected to increase visibility and impact of the journal, stimulate its growth, and strengthen its reputation within the community.

Online publishing offers many practical advantages beyond electronic handling of manuscripts right up to the point of publication. Online versions of most other journals are bound by the need for consistency with a printed equivalent. *Geochemical Transactions*, as in the past, supports the use of color to create high-quality figures [9] and present color images [10], hyperlinking [11], video files [12], and other options not available in print-only media. Use of color and other available options is not required, but they do provide authors a high degree of flexibility in the presentation of their research. Additional information such as extensive experimental data sets, model input and output files, spectroscopic data, and other relevant supplemental materials that assist readers will continue to be provided. For example, in one of our first papers, the authors were able to embed machine-readable MS and NMR data files within the paper [13]. This allows readers to directly compare their own results to those published in *Geochemical Transactions*. As demonstrated in our first five years, the online-only publishing format can add significant functionality to your publication. Moreover, as an online-only journal, *Geochemical Transactions *is in an excellent position to meet your needs and requirements for data sharing and archiving.

The scope of the journal remains unchanged. Geochemical research can be broadly defined as the study of chemical processes in earth and planetary environments. Geochemical research is pursued from the nanoscale to the planetary scale and covers both ancient and modern systems. The breath of the subject area has made this subdiscipline into a highly multidisciplinary endeavor. Within the last two decades, geochemists and scientists in affiliated disciplines have not only contributed to our understanding of geological processes through space and time but also to a wide range of other research topics. For example, geochemists have contributed to our knowledge of the fate and transport of contaminants in the environment, the effect of human activity on global systems, the role of microorganisms in the environment, the origin and evolution of life, and the processes that have shaped the surface of Mars. As part of their research effort, geochemists have developed or improved analytical tools to study the complex materials they work with to unravel the processes of interest. Complementary to analytical and experimental approaches, geochemists have developed and used theoretical models to pursue their research. *Geochemical Transactions *will continue to accept submissions of research articles, methodology, and review articles that cover this broad field of research.

We will continue our rigorous peer review system that has been established from the outset. Our expanded Editorial Advisory Board will assist us in maintaining the high quality of the journal's output our community has come to expect.

We are excited about the future of *Geochemical Transactions *as an open access journal.

Martin Schoonen, Ken Anderson, and Scott Wood

Editors-in-Chief
